# The Role of CA‐125 in the Management of Ovarian Cancer: A Systematic Review

**DOI:** 10.1002/cnr2.70142

**Published:** 2025-03-11

**Authors:** Zohre Momenimovahed, Afrooz Mazidimoradi, Leila Allahqoli, Hamid Salehiniya

**Affiliations:** ^1^ Reproductive Health Department Qom University of Medical Sciences Qom Iran; ^2^ Shiraz University of Medical Sciences Shiraz Iran; ^3^ Midwifery Department Ministry of Health and Medical Education Tehran Iran; ^4^ Department of Epidemiology and Biostatistics, School of Health, Social Determinants of Health Research Center Birjand University of Medical Sciences Birjand Iran

**Keywords:** CA‐125 antigen, disease management, ovarian cancer

## Abstract

**Background:**

Ovarian cancer is frequently occurring and fatal for women. CA‐125 is important in the screening, diagnosis, and treatment of ovarian cancer. This review study was conducted to explore the influence of CA‐125 in addressing ovarian cancer.

**Methods:**

To investigate the role of CA‐125 in ovarian cancer, we conducted a comprehensive search for high‐quality articles in the Medline, Web of Science Core Collection and Scopus databases using the keywords “ovarian cancer,” “ovarian carcinoma,” “ovarian neoplasms,” and “CA‐125” from the 2000 to 2024. We included full‐text, peer‐reviewed articles in English with relevant keywords published since 2000. We excluded case reports, commentaries, letters to the editor, books, case series, systematic reviews, animal studies, and articles that were not accessible in full text.

**Results:**

After screening the 7947 records, 88 studies were included in this review. In the literature review, it was found that researchers utilized CA‐125 for diagnosing ovarian cancer, its predicting, evaluating treatment response, assessing ovarian cancer survival, and early detection of recurrence. In some cases, researchers employed additional tumor markers alongside CA‐125 to enhance the test's sensitivity.

**Conclusion:**

CA‐125 has become a pivotal marker for ovarian cancer. Its role in the diagnosis, treatment, and ongoing assessment of ovarian cancer cannot be overstated. Continuous monitoring of CA‐125 levels can provide comprehensive insights, and categorizing patients as low‐risk or high‐risk based on CA‐125 levels could lead to better outcomes. Integrating CA‐125 with other biomarkers may enhance the accuracy of the test and elevate its relevance in patient care.

## 1 Introduction

In 2019, the global impact of ovarian cancer raised serious concerns as 294 422 new cases were reported, resulting in 198 412 deaths [1]. Over the past three decades, the prevalence of ovarian cancer has notably increased, almost doubling in incidence. This rise has been closely linked to changes in lifestyle [1]. Factors such as the adoption of Western habits, including trends in fertility, later marriage, reduced interest in childbearing, and decreased breastfeeding, have played a significant role in driving this escalation [2, 3, 4].

Epithelial ovarian cancers make up about 90% of all ovarian cancer cases, while nonepithelial ovarian cancers, which include germ cell tumors and sex cord‐stromal tumors, represent approximately 10% of ovarian cancers [5]. Ovarian cancer is often difficult to detect because it does not typically present with clear symptoms. Currently, there are no effective tools for screening the general population [6]. This frequently leads to diagnoses at advanced stages, resulting in low 5‐year survival rates [7]. It is important to note that early‐stage treatments are effective, underscoring the significance of early detection in improving survival rates. Therefore, improving diagnostic techniques, identifying high‐risk individuals through screening, and increasing awareness are crucial steps in achieving early detection and decreasing ovarian cancer mortality rates [4, 8].

Patients with ovarian cancer are faced with high treatment costs. Therefore, cost‐effective strategies for the early detection and prevention of ovarian cancer have been investigated over the last decade [6]. There has been a significant focus in recent years on finding reliable diagnostic biomarkers for ovarian cancer. Proteomics technologies like mass spectrometry and protein array analysis have advanced our understanding of ovarian cancer's molecular signaling and response to therapy. Analyzing these factors (such as PI3K upregulation in epithelial ovarian cancer) can reveal new therapeutic options, potentially reducing drug resistance and improving patient outcomes [9, 10]. Biomarkers associated with tumors have become essential for diagnosing and treating ovarian cancer [11]. There are numerous biomarkers being studied [12]. Among these, CA‐125, also known as carbohydrate antigen 125, has stood out as a particularly crucial factor in the screening, diagnosis, and management of ovarian cancer in recent years [13].

Extensive research conducted globally has emphasized the pivotal role of CA‐125 in the fight against ovarian cancer [14, 15]. Comprehensive review studies play a critical role in shaping the development of programs and the establishment of policies by providing deep insights into different facets of a subject. Consequently, this review study was initiated to investigate the impact of CA‐125 on the worldwide approaches to tackling ovarian cancer.

## 2 Materials and Methods

### 2.1 Search Strategy

In order to investigate the role of CA‐125 in ovarian cancer, a thorough search for high‐quality articles was conducted by H.S in the Medline, Web of Science Core Collection (including indexes such as SCI‐EXPANDED, SSCI), and Scopus databases using the keywords “ovarian cancer,” “ovarian carcinoma,” “ovarian neoplasms,” and “CA‐125,” both individually and in various combinations from the year 2000 to 2024. All keywords were cross‐referenced with the PubMed Medical Subject Heading (MeSH). Additionally, in order to find relevant research, we conducted searches for reference lists of similar systematic review articles, as well as articles that cited these reviews. All retrieved articles were cataloged in Endnote X7. Throughout this process, the PRISMA statement and the guidelines recommended by Moher et al. [16] were followed.

### 2.2 Data Extraction

In the study, two reviewers worked independently to gather data (Z.M, H.S). If there were any discrepancies between the two reviewers, they discussed and debated the issues until they reached an agreement. This process helped to resolve any differences in data extraction and ensured that the findings were interpreted in a consistent manner.

### 2.3 Inclusion Criteria

In order to select articles for the study, two researchers independently reviewed the retrieved articles in a rigorous two‐step screening process. Only articles that met specific criteria were included in the study. These criteria included being full‐text quantitative articles that have been peer‐reviewed, are in English, contain relevant keywords in the title, abstract, or keywords, and have been published since 2000. Articles that mentioned the role of CA‐125 in different aspects of ovarian cancer management were included in the study.

### 2.4 Exclusion Criteria

The study excluded certain types of articles based on the following criteria: case reports, commentaries, letters to the editor, books, case series, systematic reviews, animal studies, and articles that were not accessible in full text.

### 2.5 Ethical Considerations

In this particular study, the researchers demonstrated unwavering dedication to maintaining the highest ethical standards throughout the process of conducting review studies. Their commitment to ensuring accuracy and reliability was evident in every phase of data collection, analysis, and dissemination of results.

## 3 Results

### 3.1 Details of Included Studies

**FIGURE 1:**
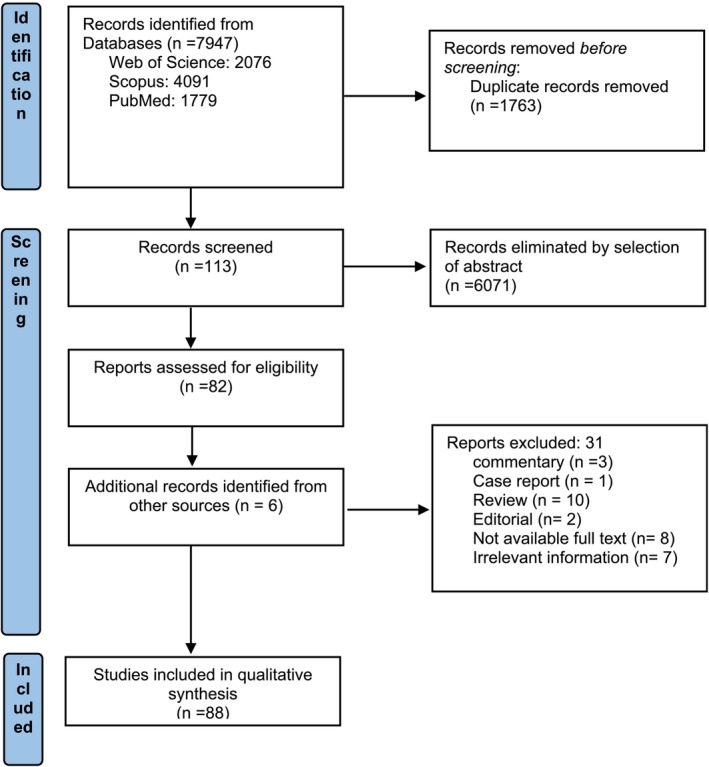
PRISMA flow diagram.

After conducting a thorough search through several databases, we initially found 7947 articles published between 2000 and 2024 for potential inclusion in our study. Using Endnote 21 software, we carefully eliminated duplicate articles, resulting in 6184 articles for further evaluation. Upon screening the titles and abstracts, we excluded 6071 articles, leaving us with 113 for a detailed full‐text review. Of these, 31 articles were excluded due to scientific considerations (Commentary: 3, Case report: 1, Review: 10, Editorial: 2, Not available full text: 8, Irrelevant information: 7). We also manually searched through relevant systematic review references and the articles they cited, and added 6 more articles. In the end, we thoroughly studied 88 articles [17, 18, 19, 20, 21, 22, 23, 24, 25, 26, 27, 28, 29, 30, 31, 32, 33, 34, 35, 36, 37, 38, 39, 40, 41, 42, 43, 44, 45, 46, 47, 48, 49, 50, 51, 52, 53, 54, 55, 56, 57, 58, 59, 60, 61, 62, 63, 64, 65, 66, 67, 68, 69, 70, 71, 72, 73, 74, 75, 76, 77, 78, 79, 80, 81, 82, 83, 84, 85, 86, 87, 88, 89, 90, 91, 92, 93, 94, 95, 96, 97, 98, 99, 100, 101, 102, 103, 104] (Figure 1).

### 3.2 Various Roles of CA‐125 in Managing Ovarian Cancer

#### 3.2.1 Diagnosis of Ovarian Cancer

Studies have shown that CA‐125 plays a significant role in diagnosing ovarian cancer [59, 97, 98, 99, 100, 101, 102]. Although CA‐125 has limited sensitivity for detecting ovarian cancer in the early stages, it is still a useful serum marker for distinguishing between ovarian cancer and borderline ovarian tumor (BOT) [100], differentiating between benign tumors and borderline ovarian tumors [99], and discerning benign from malignant tumors [98]. Research indicates that this tumor marker is utilized to determine the type and stage of ovarian cancer [21, 67, 88, 93, 94, 95, 96]. In Ahmad et al.'s study, the researchers found that patients with serous subtype and stage II ovarian carcinoma had the highest levels of CA‐125, followed by patients at stages III, I, and IV [97]. This finding was also supported by Cooper et al.'s study, where they retrospectively analyzed CA‐125 levels in 142 patients with epithelial ovarian cancer and found that higher preoperative CA‐125 values were associated with serous histology, advanced stages (III and IV), higher tumor grade, and ascites [76]. However, other studies did not find a significant correlation between tumor mass volume [17] or disease stage [59] and CA‐125 levels. It was also noted that CA‐125 measurement is more useful after menopause, with higher sensitivity, specificity, and positive predictive values in this group [92].

In their study, Antovska et al. concluded that CA‐125 alone is not useful as a single diagnostic test [91]. Therefore, many researchers have added other markers to improve the diagnostic accuracy of CA‐125 [88, 89, 90]. One study found that although CA‐125 alone detected more than half of early stage ovarian cancer cases, its combination with HE4 Ag‐AAb complexes increased the detection rate to 81% [87]. Kim et al. stated that while CA‐125 has good diagnostic performance, its accuracy varies depending on the type of ovarian cancer. Therefore, they recommended that to increase accuracy, it is better to use CA‐125 in combination with HE4 and ROMA [86]. Sørensen and Mosgaard demonstrated that the CA125/CEA ratio can serve as a preoperative diagnostic tool for ovarian tumors. They found that when the CA125/CEA ratio exceeds 25, ovarian cancer is diagnosed in 82% of cases [85]. In a case–control study involving 254 healthy women and 75 breast cancer patients, Andersen et al. revealed that the combination of CA‐125 and symptom index detected cancer in 89.3% of women, 80.6% of whom had early‐stage cancer, and 95.1% of those with advanced cancers [84]. Furthermore, a study indicated that HE4 exhibited a satisfactory ability to differentiate between endometriosis and ovarian cancer, a capability that CA125 lacked [83].

#### 3.2.2 Prediction of Ovarian Cancer

The significance of CA‐125 in predicting various aspects of cancer has been extensively studied. CA‐125 levels can provide valuable insights into resectability, response to treatment [35, 79, 80, 81, 82], metastasis [78], as well as the likelihood of recurrence and mortality prior to surgery 17. Research indicates that a 5% reduction in CA‐125 levels post‐surgery is indicative of disease stability, while an increase of more than 10.5% suggests progression, warranting further diagnostic assessment such as CT scans [74]. Additionally, retrospective analysis has revealed that pre‐treatment CA‐125 levels ≥ 35 U/mL independently correlate with prognosis in ovarian cancer patients. According to recent research, patients with elevated CA‐125 levels prior to treatment should be categorized as high‐risk for relapse or mortality [73]. Another study by Wang et al. indicated that a reduction of less than 97.6% in CA125 after the fourth cycle of chemotherapy could serve as a predictive factor for relapse within 12 months. Patients who do not experience a significant decrease in CA125 after four cycles of chemotherapy should undergo regular follow‐ups and more active re‐evaluations [77]. Additionally, a retrospective study of 300 patients in the United Kingdom revealed that in cases where CA‐125 levels decrease to normal after chemotherapy, a doubling of the upper normal limit predicts disease progression [72]. Studies have shown a correlation between elevated CA‐125 levels and a reduced likelihood of achieving optimal cytoreduction [71]. Cooper et al. demonstrated that preoperative CA‐125 levels below 500 have an 82% positive predictive value and a 48% negative predictive value for optimal cytoreduction. However, another study suggested that preoperative CA‐125 may not be a reliable indicator for optimal cytoreduction [76].

According to some researchers, combining CA‐125 with other tumor markers or human tissue kallikreins is advised to enhance the prognosis of epithelial ovarian cancer [70]. Roupa et al. found that a combination of CA‐125 greater than 30 and a TVUS score of greater than or equal to 35 has a sensitivity of 81.7% and a specificity of 100% in predicting ovarian cancer [69].

#### 3.2.3 Response to the Treatment

CA‐125 serves as a potent indicator for monitoring ovarian cancer [68] and can be utilized to gauge the response to treatment [18, 19, 20, 21, 22, 23, 24] as it is the most powerful indicator. A study revealed that the levels of CA125 become negative in the fourth cycle for patients showing a positive response to chemotherapy [25]. Moreover, suboptimal cytoreduction (more than 1 cm residual) is significantly linked to elevated CA‐125 levels [26]. A study conducted in Iran indicated that the reduction of CA125 after neoadjuvant chemotherapy can forecast the clinical outcome of patients [27]. Furthermore, an investigation involving 103 patients with stage III or IV ovarian cancer demonstrated that patients with preoperative CA‐125 levels ≤ 1000 are more likely to have no remaining cancer after surgery [28]. The findings of a study revealed a correlation between CA‐125 levels of ≥ 500 U/mL and carcinomatosis of the diaphragm and residual disease [29]. However, some researchers have cautioned against solely relying on CA‐125 trends for treatment decisions as it may lead to premature discontinuation of treatment in potential responders [30]. They have also suggested that re‐evaluating CA‐125 levels in patients whose levels normalize preoperatively and after neoadjuvant chemotherapy may not be necessary [50].

#### 3.2.4 Ovarian Cancer Survival

The level of CA‐125 is a reliable indicator of survival [66, 67]. Tracking CA‐125 over time can predict both overall survival [23, 26, 33, 36, 37, 39, 51, 52, 53, 54, 55, 56, 57] and progression‐free survival [23, 33, 34, 35, 36, 37, 38, 39]. Normal pre‐treatment levels of CA‐125 are linked to improved overall survival and progression‐free survival [31]. It is recommended to monitor CA‐125 during chemotherapy, particularly in the third cycle, as changes in its levels are a valuable predictive factor. A negative CA‐125 marker after the third cycle of chemotherapy and lower‐than‐average levels at the time of diagnosis may be associated with progression‐free survival [25].

The study's findings revealed a significantly lower 5‐year survival rate in patients with a CA‐125 increase to 5 U/mL at 3‐ and 6‐months post‐treatment [32]. According to a study, preoperative serum CA‐125 levels exceeding 535 are indicative of lymph node metastasis [32]. The presence of lymph node metastasis is associated with poor prognosis in terms of progression‐free survival and overall survival [48]. Additionally, Chiang indicated that patients with low CA‐125 levels (< 35 U/mL) are more likely to successfully undergo interval debulking surgery and experience prolonged progression‐free survival compared to those with high CA‐125 levels (> 100 U/mL) [40]. In his study, Chan demonstrated that elevated CA‐125 levels prior to chemotherapy were independently linked to poorer recurrence‐free survival (HR = 2.13, 95% CI: 1.23–3.69; *p* = 0.007) and overall survival (HR = 1.99, 95% CI: 1.10–3.59; *p* = 0.022) [59]. Furthermore, the findings from a study involving 112 stage III and IV patients indicated that a 75% or greater decrease in serum CA‐125 levels from primary cytoreductive surgery to the initiation of adjuvant chemotherapy served as an independent prognostic factor for progression‐free survival in patients with stage III and IV ovarian cancer [64]. According to a study, a reduction of more than 50% in CA‐125 levels within 8 weeks post‐operation was linked to a 21‐month survival, while a reduction of less than 50% was linked to a 10‐month survival. The decrease in serum CA‐125 concentration during two cycles of platinum‐based chemotherapy is a strong independent predictor of survival in stage III or IV ovarian cancer [65].

While some researchers have demonstrated that preoperative CA‐125 levels do not serve as predictors of recurrence‐free survival [59, 60, 61, 62, 63] and that postoperative reduction is also not indicative of outcome [49], it has been found that an increase in CA‐125 (≥ 35 U/mL) after completing primary treatment is linked to poorer progression‐free survival and overall survival [60].

#### 3.2.5 Early Detection of Recurrence

Baseline CA‐125 levels before starting maintenance chemotherapy strongly correlate with the risk of subsequent recurrence [58]. Serial monitoring of CA‐125 levels can lead to early detection of recurrence, potentially improving survival [48]. Additionally, elevated serum CA‐125 levels serve as an early indicator of clinical recurrence in women with ovarian cancer [32, 40, 41, 42, 43]. A study demonstrated that a gradual increase in CA‐125 levels in patients with epithelial ovarian cancer during clinical remission predicts disease recurrence. Specifically, a relative increase in CA‐125 levels of 100% was significantly predictive of recurrence [44].

In a recent retrospective study involving 99 patients with recurrent epithelial cancer, findings suggested that there is a heightened probability of extra‐pelvic recurrence and multiple recurrences as CA‐125 levels increase [46]. Research indicates that a progressive increase in CA‐125 levels within the normal range at 1–3 months is linked to an elevated likelihood of recurrence. As a result, patients exhibiting this pattern should undergo immediate evaluation to either rule out or detect cancer recurrence [47]. Furthermore, in patients with advanced epithelial cancer who are in complete remission, a slight increase in CA‐125 levels within the normal range independently predicts disease recurrence [45].

Dondi et al. found a significant association between CA‐125 levels and PET/CT results [104]. However, Wang demonstrated that the early detection rate of recurrence using CA‐125 levels was just 13% [103]. Another study concluded that neither the pre‐surgery CA‐125 concentration nor its reduction was correlated with prognosis for recurrence and survival at any stage [35].

## 4 Discussion

Ovarian cancer is both common and unfortunately one of the deadliest forms of cancer worldwide [4]. Type II epithelial ovarian cancers are thought to be biologically aggressive tumors from their onset, displaying a tendency for metastasis even from small primary lesions. In contrast, type I epithelial ovarian cancers are considered to be relatively indolent and genetically stable, typically arising from identifiable precursor lesions such as endometriosis or borderline tumors of low malignant potential [105]. In recent years, CA125 has played a crucial role in diagnosing, predicting outcomes, and treating ovarian cancer, making it one of the most commonly utilized biomarkers to date [106]. In a clinical setting, CA‐125 serves several important purposes. For postmenopausal patients with a suspected pelvic mass, elevated CA‐125 levels increase the probability of ovarian cancer, prompting a referral to an oncologist for optimal care. Furthermore, a decrease in CA‐125 levels during chemotherapy provides reassurance to both the doctor and the patient that the tumor is responding to treatment. Conversely, an increase in CA‐125 levels during chemotherapy may indicate the development of drug resistance, necessitating a change in or cessation of ineffective treatment. Lastly, a high CA‐125 level at the conclusion of six planned chemotherapy cycles suggests the presence of residual disease, prompting proactive measures [107].

Due to the high rates of false positives and false negatives, CA125 does not seem to be a reliable biomarker for diagnosis, nor does it lead to a reduction in mortality when used as a diagnostic index. About one third of women suffering from non‐gynecological cancers and various non‐oncological conditions exhibited elevated levels of CA‐125 [108]. As a result, various strategies have recently been suggested to enhance the reliability of CA125, such as combining it with other biomarkers and measuring glycovariants [13]. According to the findings of the current study, despite the limited sensitivity of CA‐125, many researchers continue to utilize it as a standalone diagnostic tool for ovarian cancer or in conjunction with other markers. Various studies have indicated that CA‐125, with a sensitivity of 80% and a specificity of 75%, is employed in clinical settings to identify malignant ovarian masses [109]. However, elevated CA‐125 levels can also be present in benign conditions such as endometriosis, fibroids, pelvic inflammatory disease, pregnancy, as well as non‐gynecological conditions like peritonitis, pleurisy, pericarditis, or non‐gynecological malignancies from the digestive system or breast, particularly in the presence of intraperitoneal metastasis, which may restrict the test's diagnostic utility [110]. A systematic review of 70 studies comprising 2374 samples revealed that CA‐125 is a valuable preoperative test for predicting benign or malignant pelvic masses [109]. The study also demonstrated that changes in CA‐125 levels can be utilized to assess treatment response. Nonetheless, a review of 2192 patients across 14 studies indicates that preoperative serum CA‐125 levels cannot predict the success of optimal cytoreduction in advanced ovarian cancer patients. Given the low positive likelihood ratio and high negative likelihood ratio, CA‐125 levels may not be reliable for confirming or ruling out the failure of optimal cytoreduction. Therefore, making clinical decisions, such as recommending neoadjuvant chemotherapy solely based on CA‐125 levels, may be inappropriate, as suggested by the findings of this study [111].

The CA‐125 peptide epitope of mucin MUC16 is known to bind to mesothelin with high affinity, promoting the attachment of cancer cells to the mesothelial lining and leading to peritoneal metastasis [112]. Wang et al., in their meta‐analysis, found that elevated CA‐125 levels before treatment are associated with poorer overall survival and progression‐free survival [112]. The study's findings suggest that the serum level of CA‐125 before treatment, regardless of tumor stage or use of NACT, holds predictive significance for prognosis [112]. Karamouza et al. found that in patients newly diagnosed with FIGO stage II to IV ovarian cancer, serum CA‐125 levels at 3 months after completing primary treatment were the most accurate indicators of overall survival at 24, 36, and 48 months. According to their research, a CA‐125 level above 35 at the 3‐month mark suggests the need for more rigorous monitoring, including regular CA‐125 concentration measurements and CT scans, to assess the potential necessity of modifying treatment [113].

The findings of the current study indicate that CA125 can be utilized in monitoring patients during their follow‐up period. Pignata et al. have noted that even though early chemotherapy treatment for asymptomatic patients does not significantly extend survival based solely on CA125 elevation, monitoring CA125 levels should still be recommended for patients during their follow‐up. Asymptomatic patients with elevated CA125 levels require more effective treatments to enhance survival and should undergo regular monitoring. These researchers are of the opinion that while regular CA125 measurements undoubtedly detect relapse before symptoms manifest in most patients, early intervention with existing medications does not contribute to improved overall survival (OS). Nevertheless, early intervention in certain patients may delay the onset of cancer‐related symptoms, such as ascites or intestinal obstruction, and enhance their quality of life [114].

CA125 and HE4 are currently the only approved biomarkers for epithelial ovarian cancer; however, their effectiveness for early detection remains limited. To address the shortcomings of single serum biomarkers, multivariate index (MVI) assays have been developed, particularly to assist in the pre‐surgical evaluation of adnexal masses. The Risk of Malignancy Algorithm (ROMA) combines menopausal status with CA125 and HE4 concentrations to aid in diagnosing women with a pelvic mass. Additionally, miRNAs show significant potential in various aspects of predicting epithelial ovarian cancer. Nevertheless, further research is necessary to characterize miRNAs as reliable biomarkers [115].

### 4.1 Limitations

The use of English‐language articles may limit the study results by excluding important data from other languages. However, due to the wide range of articles in the target area, qualitative evaluation of the articles was not conducted, and all studies meeting the inclusion criteria were included in the study. The study has limitations such as heterogeneity in study designs, limited sample sizes in some studies, and different characteristics of patients and control groups. These limitations may impact the accuracy of data analysis and the generalization of findings. Therefore, caution should be exercised when generalizing the findings, and it is important to recognize these limitations and potential variability in the data. Nevertheless, the systematic and comprehensive review of the role of CA‐125 in the management of ovarian cancer which was conducted independently by two investigators provides valuable information to the reader. The dual screening of articles significantly enhanced the quality of the report.

## 5 Conclusions

Early detection of ovarian cancer is crucial for improving long‐term prognosis. CA‐125, has become a pivotal marker for ovarian cancer over the last few decades. Its pivotal role in the diagnosis, treatment, and ongoing assessment of ovarian cancer cannot be overstated. Continuous monitoring of CA‐125 levels can provide researchers and healthcare providers with more comprehensive insights compared to individual measurements. Categorizing patients as low‐risk or high‐risk based on CA‐125 levels, and closely tracking high‐risk patients, could lead to better outcomes. Additionally, integrating CA‐125 with other biomarkers may enhance the accuracy of the test and elevate its relevance in patient care.

## Author Contributions

All authors made contributions to all sections of the writing and collectively approved the final submitted version of the article.

## Conflicts of Interest

The authors declare no conflicts of interest.

## Data Availability

Data sharing is not applicable to this article as no new data were created or analyzed in this study.
